# Co Treatment With Biologic Agents and Immunotherapy in the Setting of irAEs of Difficult Management

**DOI:** 10.3389/fmed.2022.906098

**Published:** 2022-06-30

**Authors:** Virginia Robles-Alonso, Fernando Martínez-Valle, Natalia Borruel

**Affiliations:** ^1^Crohn's and Colitis Attention Unit, Digestive System Service, Hospital Vall d'Hebron, Barcelona, Spain; ^2^Systemic Autoimmune Diseases Unit, Internal Medicine Department, Hospital Universitari Vall d'Hebron, Barcelona, Spain; ^3^Centro de Investigaciones Biomédicas en Red de Enfermedades Hepáticas y Digestivas (CIBEREHD), Madrid, Spain

**Keywords:** immunotherapy, adverse drugs reaction, immunosuppression therapy, immune check-point inhibitors therapy, autoimmune diseases–therapy

## Abstract

In recent years, immunotherapy has become an important pillar of cancer treatment, with high response rates regardless of tumor histology or baseline mutations, sometime in patients without any alternative of treatment. Moreover, these treatments are moving from later line therapies to front-line therapies in the metastasic setting. However, immune activation associated with immune check-point inhibitors (ICI) is not selective and a large variety of immune-related adverse events, with an increasing frequency, have been associated with anti-PD1, anti-PD-1/L-1 and anti-CTLA-4 agents. In clinical trials, and sometimes also in real life practice, patients who develop severe toxicities on ICI-based therapies are usually not allowed to resume ICI once their disease progresses, because of the chance of developing severe irAEs on rechallenge with immunotherapies. Moreover, patients with irAEs suffer important side effects due to the high dose corticosteroids that are used to treat them. Therapy with ICI is sometimes the only alternative for certain patients, and for this reason co treatment with classic (DMARDS) or biologic immunosuppression therapy and ICI must be considered. Co-treatment with this type of immunosuppressant drugs, apart from allowing the maintenance of ICI therapy, drive to a lesser use of corticosteroids, with an improvement of the safety and quality of life of the patients. Such a tailored scheme of treatment is mostly an expert opinion based on recommendation and currently there is scarce evidence supporting it. Herein we present comprehensive, current recommendations and real-world data on the use of co-treatment with ICI and DMARDS and biologic immunosuppression.

## Introduction

Immunotherapy has become an important pillar of cancer care, complementing surgery, cytotoxic therapy and radiotherapy in most tumor types. Description of immune-editing by Schreiber (1) as a process that enables escape from immune surveillance to establish overt malignancy and characterization of cancer-immunity cycle by Chen and Mellman (2) impacted on the development of multiple opportunities for therapeutic intervention enhancing tumor immunity. Immune check-point inhibitors (ICI) that target the programmed death protein 1 pathway (anti-PD-1: nivolumab and pembrolizumab) and its ligand (anti-PD-L1: atezolizumab, avelumab and durvalumab) have obtained the most impacting outcomes with response rates across tumor types of 20–30%. The other group of ICI, anti-cytotoxic T-lymphocyte-associated antigen drugs (anti-CTLA-4: ipilimumab and tremelimumab) engage T cells with inherent capacity for adaptability and memory, that leads to durable responses and long-term survival.

The safety profile of ICI differs from chemotherapy or targeted therapy since immune-related adverse events (irAEs) result from immune activation driving autoimmune manifestations. Overall, the majority of patients treated with ICI developed some irAEs, although the rate of grade 3 events is low (around 10%), except for patients treated with ICI combination. Immune-related adverse events usually present within the first weeks of ICI therapy, though they can occur anytime. In clinical trials, and sometimes also in real life practice, patients who develop severe toxicities on ICI-based therapies are usually not allowed to resume ICI once their disease progresses, because of the chance of developing severe irAEs on rechallenge with immunotherapies. Moreover, patients with irAEs have relevant side effects due to the high dose of corticosteroids that are used to treat them. Not only rapid resolution of irAEs is required, but prevention of irAE recurrence from re-exposure to ICI is also mandatory. Therapy with ICI is sometimes the only alternative for some patients, and therefore co-treatment with classic (DMARDS) or biologic immunosuppression therapy and ICI must be considered. In this way, the use of biologic immunosuppression with cytokine inhibitors usually offers a quicker response in front of DMARDS. Unlike corticosterois and classic DMARDS, which inhibit several inflammatory processes in an unspecific way, cytokine inhibitors provide a targeted clinical approach to reduced ICI-induced inflammation. This fact underlines the need for appropriate therapeutic selection based on a mechanistic understanding of the differential immune conditions that drive the different irAEs (3). Moreover, in the selection of the immunosuppressant agent for a given irAE it is important to take into consideration the current standard treatment for similar non-ICI related conditions. Co-treatment with these types of immunosuppressant drugs, apart from allowing maintenance of ICI therapy leads to a lesser use of corticosteroids and thus, to an improvement in patient safety and quality of life. On the other hand, the co-administration of immunosuppression in the treatment of irAEs has potentially both advantages and disadvantages, given their potential to impact over multiple aspects of the immune system, including infection and antitumour immunity. As an example, there is evidence that an anti-IL 17 antibody, secukinumab, can impair the effect of pembrolizumab in colon rectal cancer (4), or the deleterious effect on the oncological outcome in retrospective studies of DMARDs in patients with immune-related arthritis (5, 6). For all these reasons, at this time a tailored scheme of treatment is mostly an expert opinion based on recommendation, currently with scarce evidence supporting it. Herein we present a comprehensive summary of current recommendations and real-world data on the use of co treatment with ICI and DMARDS/biologic immunosuppression.

## Scenario for Concurrent Immunosuppression and Rationale Basis

The majority of patients with irAEs will respond to corticosteroids, but a small group of them will require immunosuppressant or biological therapy for corticosteroid dependency or refractoriness. Moreover, in many patients with ICI-induced irAES it might be necessary to maintain immunotherapy, even indefinitely, to achieve or sustain underlying tumor remission. However, the scenario of a patient with moderate to severe irAEs but favorable tumor response to immunotherapy raises doubts about the risk of resuming immunotherapy again. This setting, positioned out of practical guidelines, is complex and depends on multiple factors like subsequent options of oncological treatment, severity and response to treatment of the ICI toxicity and coexistence of other immune-mediated diseases (IMID). Although the final decision in this clinical scenario will depend on the oncologist, it should be endorsed by a panel of different specialist that play a crucial role in establishing a therapeutic strategy in case of resuming immunotherapy. Given the lack of prospective clinical trials, the final decision usually is based on expert opinion and evidence available up to now. In a very interesting recent paper, Haanen (7) propose three possible options of retreatment in case of previous severe toxicity: class switch, rechallenge, and resumption with concurrent immunosuppression.

Regarding the use of ICI with a simultaneous immunosuppression there are limited data available apart from published reports, but it may be the best option for those patients with severe irAEs, mostly in the absence of therapeutic alternatives (7). After a high grade irAEs it is challenging the ICI resumption because of the risk for recurrence and the absence of guidelines. Ideally, the selection of the concurrent agent should be based on the irAE type, response to immunosuppression, life expectancy, quality of life, comorbid conditions and patient preferences.

The basis of theoretical rationale to use cotreatment relies on different relevant arguments. In the first place, there is some evidence that blockade of some endogenous cytokines by monoclonal antibodies can confer anti-tumoral properties. In different clinical situations, second-line immunosuppressant treatment for irAEs frequently includes anti-TNF biological therapy. Some recent evidence shows that blocking TNF alfa, a cytokine with broad well-known pleiotropic effects, before combination therapy with anti-CTLA-4 and anti-PD-1 agents in tumor-bearing mice, would not only prevent autoimmune toxicity but also stimulate anti-tumoral efficacy (8, 9). The underlying mechanism would be the capability of TNF to stimulate activation-induced cell death (AICD) of CD8+ T cells impairing their accumulation in tumors and consequently promoting tumor growth and impeding response to anti-PD-1. This evidence has settled the basis to carry out the TICIMEL study in humans (clinical trials.gov id: NCT03293784), a phase-1b clinical trial in which Nivolumab and Ipilimumab are administered in combination with Infliximab or Certolizumab (antiTNF antibodies) in patients with advanced melanoma. On the other hand, interleukin-6 can promote tumor progression and metastasis by activation of several oncogenic pathways, increase survival of myeloid derived suppressor cells and inhibition of dendritic cell differentiation (10). Moreover, the IL-6/JAK/STAT3 pathway plays a role in the generation of an inflammatory response that is responsible for many symptoms associated to cancer, like the impairment of the quality of life or the performance status (11). Furthermore, the upregulation of the IL-6 pathway associated with a sustained chronic inflammation may hamper ICI efficacy and worsen the prognosis of the oncologic disease (12). Some reports have linked an increased level of circulating IL-6 with some irAEs like cholangiohepatitis and pneumonitis, and in these settings, the treatment with tocilizumab, a specific IL-6 receptor inhibitor, has been reported effective (13, 14). In the same way, it has demonstrated efficacy in the treatment of cachexia associated with cancer (15).

Additionally, although many immune-related adverse events (irAEs) respond to corticosteroids, a significant number of patients develop corticosteroid dependency or refractoriness. In this subgroup of patients, a corticosteroid-sparing strategy could avoid unnecessary and deleterious side effects. In checkpoint inhibitor-associated colitis there are some factors, like the presence of deep ulcers in the colonic mucosa, that predict those patients at a higher risk of steroid-refractory behavior. In addition, a retrospective study from Abu-Sbeih et al. (16) demonstrates that those patients with ICI–induced colitis who start immunosuppressive therapy earlier (<10 days after colitis onset vs. >10 days) have better outcomes in terms of fewer hospitalizations, a shorter duration of symptoms and less use of corticosteroids.

Another argument to indicate combination therapy in patients with previous irAEs is based on the fact that, in specific advanced tumors, better response rates and survival outcomes were obtained among patients who developed any irAE of any severity as compared to those who did not. Similar results were reproduced by different retrospective analyses both in advanced melanoma and NSCLC (17–19). Another interesting study by Naqash (20) analyzed data from 531 metastatic NSCLC (non-small cell lung cancer) treated with nivolumab after non-response to first line therapy. Thirty-three percent of patients who developed irAE had significantly better outcomes in terms of survival as compared to those who did not develop any irAE. A retrospective analysis from the prospective nationwide Dutch Melanoma Treatment Registry (21) explored the association between severe toxicity development and overall survival. Thousand two hundred fifty patients were included, 25% of whom suffered severe toxicity (> = 3), and showed a better survival than those who did not (23 vs. 15 months).

Furthermore, it is also known that immunotherapy discontinuation due to irAE has worse results in terms of survival. Santini et al. (22), in a study with patients with advanced NSCLC treated with anti–PD-L1 who stopped it due to irAEs divided these patients into two groups: those retreated with anti–PD-L1 (retreatment cohort) or those who had treatment stopped (discontinuation cohort). Among those patients with no observed partial responses prior to the irAE, survival outcomes were better in the retreatment cohort. Conversely, for those with objective responses prior to the irAE, survival outcomes were similar in the retreatment and discontinuation cohorts. These results suggest that retreatment, especially in patients with irAEs who had no treatment response prior to irAE onset, could be beneficial in terms of tumor response and survival. However, prospective studies with more patients included would be necessary to validate this data.

## Real World Data of Concurrent Immunosuppression

A myriad of case reports of irAEs treated with anti-citokine monoclonal antibodies have been described. Badran et al., (23) described a five-patient case series with different primary tumors who developed gastrointestinal immune-related adverse events, all of them with moderate to severe upper and/or lower gastrointestinal endoscopic lesions. Three out of four developed corticoid-dependency or refractory behavior. All of them received cotreatment with immunotherapy and infliximab over a period ranging from 4 to 10.5 months without tumoral progression or even with improvement in all but one. Regarding GI toxicity, patients remained asymptomatic or with mild symptoms despite ongoing immunotherapy. Another strategy of treatment for immune mediated colitis is the use of vedolizumab, an α4β7 integrin inhibitor that blocks T cells trafficking to the gut, and that is used frequently in the setting of inflammatory bowel disease. Vedolizumab has been used concomitantly when therapy with ICI is restarted after the resolution of immune mediated colitis (16). With this cotreatment, only one out of eight patients presented a recurrence of digestive manifestations.

Another publication from Kim (24) reports three more cases of cotreatment. In one case, a patient who developed an immunomediated arthritis with corticoid dependency and a chronic course, was successfully treated with tocilizumab. The patient remained in complete remission, although immunotherapy was not resumed after receiving tocilizumab. In a second patient, cotreatment with tocilizumab and a non-concrete investigational melanoma therapy for over 15 months, controlled irAE corticodependent arthritis. A third case presented a patient with a non-specified severity colitis and arthritis, treated in combination with ipilimumab and tocilizumab for over 3 months. Both toxicities were kept under remission although with a demonstrated tumor progression. Stroud et al., (25) analyze the use of tocilizumab in a wide variety of irAEs in a single center study. Among the 87 patients who received treatment with nivolumab, 34 (39.1%) required treatment with tocilizumb due to the presence of a wide range of steroid refractory irAEs, including pneumonitis, systemic inflammatory response, cerebritis, hypophysitis, colitis, pancreatitis and hepatitis. Clinical improvement was noted in 79.4% of patients, and in 47% of them more than one dose was required. In a systematic review about the use of tocilizumab, Champochiaro et al. (26), reported that in 85% of the 91 patients in whom this drug was used, a clinical benefit was observed, without any case of disease progression, and for that reason, the use of tocilizumab may be a safe alternative for long treatments.

While no solid conclusions can be drawn from small series of cases, it generates enough evidence to develop clinical trials and consider cotreatment in specific clinical scenarios.

## Safety of Concurrent Immunosuppression

Another aspect of concern when introducing cotreatment therapeutic strategy would be safety issues. Since current recommended strategies do not consider this approach, information can only be gathered from indirect studies. Recent descriptions of the role of TNFα in tumor biology has supported the concurrent immunosuppression with anti-TNF molecules. TNFα produced in the setting of anti-PD-1 blockage leads to an impairment in the CD8+ tumor infiltrating T lymphocyte responses (27). On the other hand, TNFα enhances activation-induced cell death in T cells, that will reduce their viability in the tumor microenvironment (28). For all these reasons by blocking TNFα both studies showed an increase in CD8+ T cell numbers and viability in the tumor microenvironment and draining lymph nodes (28). In this regard, Lesage et al., (29) conducted a retrospective study in order to measure the impact of antiTNF treatment on disease outcome in advanced melanoma patients. Twenty-seven patients with ICI grade 3/4 induced colitis and subsequently treated with antiTNF were included. The overall survival, progression-free survival and objective response rate were compared with those reported in pivotal studies, concluding that neither the occurrence of colitis, nor antiTNF treatment seemed to affect disease outcomes. Weber and colleagues (30) reported GI toxicity occurrence and its management, among patients receiving ipilimumab and nivolumab from two randomized trials. In 22 patients with ICI induced colitis that received steroids along with anti TNF antibodies, there were no differences in tumor response rates and survival as compared to those that received steroids alone. Similarly, Johnson et al., (31) reported no differences in overall survival in 40 patients treated with ICI who developed grade 2–4 colitis and received either high dose steroids or steroids in combination with anti TNF alpha.

A retrospective analysis from the prospective nationwide Dutch Melanoma Treatment Registry (21) explored the association between severe toxicity development and overall survival. Twenty-five percent of the 1,250 patients included suffered severe toxicity (≥3), showing a better overall survival than those who did not (23 vs. 15 months). In contrast to other studies, in this group of patients experiencing severe toxicity, those who received anti-TNF had worse survival outcomes than those receiving corticoids alone. The authors suggest that TNF-alpha blockade would abolish the survival advantage associated with toxicity. Other reasons advocated to explain such discrepancies could be related to different efficacy outcomes measurement and immortal time bias (32).

We should take into account that vedolizumab, due to its mechanism of action by hampering T cell trafficking into the gut, is not recommended in primary gastrointestinal tumors.

Bearing in mind the paucity of solid evidence and clinical experience, cotreatment implies a certain degree of uncertainty. However, in view of the current data available, it seems reasonable to use longstanding cotreatment to prevent flares of irAEs with vedolizumab or adalimumab in ICI-induced enterocolitis, and tocilizumab in ICI-induced inflammatory arthritis.

In view of the uncertainty of the current knowledge, it seems reasonable to undertake prospectively designed studies to assess the relationship of overall outcomes not only with the severity of the irAEs, but also their location, and with the administered treatment.

## Coexistence of Autoimmune Diseases

A specific sub-population to take into account when considering cotreatment, are patients with a previous history of autoimmune disease (IMID). These patients have been traditionally excluded from clinical trials so it has been necessary to analyse some retrospective studies to obtain a comprehensive view. Versphol et al., in a single center study involving a large series of patients treated with ICI (33), described that one-third of patients with pre-existing rheumatic disease experienced a disease flare, but in none of them did ICI therapy have to be stopped. Moreoever, no new new rheumatological diseases appeared in these patients. Menzies (34) assessed another cohort of patients with previous history of rheumatoid arthritis, polymyalgia rheumatica, Sjogren's syndrome, thrombocytopaenic purpura, and psoriasis. Twenty-nine percent of them developed irAEs motivating discontinuation of treatment in 8% of them. Another remarkable report (35) explores safety and efficacy of ipilimumab in 30 patients with pre-existing autoimmune disorders. At the time of ipilimumab treatment initiation, 13 patients (43%) were on treatment with at least 1 systemic therapy (6 receiving low-dose steroids, 5 hydroxy-chloroquine sulfate, 1 leflunomide, and 1 methotrexate). Twenty-seven percent of patients had some type of exacerbation of their autoimmune disease that required treatment with 10 patients (33%) experiencing grade 3 to 5 irAEs. A proposed therapeutic strategy in these patients would be to evaluate IMID activity and severity behavior before immunotherapy onset. In a recent study from Abu-Sbeih (36), patients with underlying IBD who needed to be treated with immune checkpoint inhibitors were retrospectively analyzed in order to describe occurrence of irAE. One hundred and two patients were included, 41% of them developed irAE and 21% of them a grade 3–4 colitis. It is also worthy of note that four patients suffered a colonic perforation, 2 of whom required surgery. Regarding therapy, it is noteworthy that 42% of patients were not receiving treatment for the underlying IBD in the 3 months before immunotherapy initiation and that 29% of patients required treatment with infliximab or vedolizumab as part of the treatment for the irAE. Another review with meta-analysis from Meserve (37) draws similar conclusions.

In patients with pre-existing autoimmune conditions it is of paramount importance to diagnose a disease flare in time. Differentiation of an ICI mediated flare of disease and a flare which would have occurred without ICI is sometimes impossible. Afterwards, in the event of an irAEs appearing, combination therapy with anti-cytokine drugs plus immunotherapy could be a treatment option.

## Discussion

Cancer immunotherapy has become one of the major breakthroughs in medical evolution that has changed the fight against cancer. However, severe toxicities associated with this type of treatment can sometimes limit its use. Management of severe irAEs can be challenging and most times are based on expert consensus or personal viewpoint due to scarce evidence until now. Co-treatment with anti-cytokine therapy, that is normally used in autoimmune conditions, and ICI has become one of most frequent strategies in the management of irAEs. Cytokine targeted therapies can provide long-term control of irAEs, even with rechallenge of CPI treatment. However, is necessary to conduct prospective investigations on side-effect management of ICI therapy in future advanced-phase trials. Moreover, proper management of severe irAEs requires the intervention of a multidisciplinary team with experience in autoimmune conditions.

The use of immunomodulatory agents in this clinical setting is based not only on the knowledge of the mediators that are involved in the development of these manifestations, mostly TNFα and IL-6, but also on the current standardized treatments of the primary autoimmune conditions that irAEs can mimic. In this review, we take into consideration some of the real word evidence.

In order to synthetize and distill all the above information, it is important to put the focus on the most relevant clinical scenarios that are outside the clinical guidelines. First of all, those patients with moderate to severe toxicity together with a consistent favorable tumor response to immunotherapy, obviously excluding those with life-threating irAE such as myocarditis, pneumonitis, or encephalitis that contraindicate resuming ICI for life. In the second place, those patients with previous IMIDs history. A feasible therapeutic approach in these challenging scenarios could be cotreatment with second-line anti-cytokine immunomodulators depending on the organ affected by the irAE, the predominant type of cytokine involved or the primary autoimmune disease that such irAEs mimic. This new strategy is supported by the evidence that better response rates and survival outcomes have been observed among patients who develop any irAE and that cotreatment with biologic agents does not seem to impair survival or oncological outcomes. However, at this time such recommendation is based only on expert opinion, and for this reason, it is fundamental to carry out prospective studies in order to clarify the best strategy for the management of these challenging manifestations.

## Author Contributions

All authors listed have made a substantial, direct, and intellectual contribution to the work and approved it for publication.

## Conflict of Interest

The authors declare that the research was conducted in the absence of any commercial or financial relationships that could be construed as a potential conflict of interest.

## Publisher's Note

All claims expressed in this article are solely those of the authors and do not necessarily represent those of their affiliated organizations, or those of the publisher, the editors and the reviewers. Any product that may be evaluated in this article, or claim that may be made by its manufacturer, is not guaranteed or endorsed by the publisher.
